# Targeted probiotic therapy in irritable bowel syndrome: a clinical evaluation on *Clostridium butyricum* CBM588 and *Bifidobacterium longum* W11

**DOI:** 10.3389/fmed.2025.1604319

**Published:** 2025-05-20

**Authors:** Alexander Bertuccioli, Davide Sisti, Nadia Lazzerini, Chiara Maria Palazzi, Giordano Bruno Zonzini, Mirko Ragazzini, Annalisa Belli

**Affiliations:** ^1^Department of Biomolecular Sciences, University of Urbino Carlo Bo, Urbino, Italy; ^2^Microbiota International Clinical Society, Torino, Italy; ^3^USL Tuscany South-East Company, Valdarno Hospital “La Gruccia”, U.O.S.D. Digestive Endoscopy, Interventional and Emergency Unit, Montevarchi, Italy

**Keywords:** irritable bowel syndrome, *Clostridium butyricum* CBM588, *Bifidobacterium longum* W11, probiotics, IBS-D, IBS-C, gut microbiota, symptom severity

## Abstract

**Background:**

Irritable Bowel Syndrome (IBS) is a chronic functional gastrointestinal disorder characterized by symptoms such as abdominal pain, bloating, and altered bowel habits. Probiotic-based strategies are increasingly being explored for IBS management, with growing interest in strain-specific applications.

**Objective:**

This study aimed to evaluate the clinical efficacy of *Clostridium butyricum* CBM588 and *Bifidobacterium longum* W11 in IBS patients with diarrhea-predominant (IBS-D) and constipation-predominant (IBS-C) symptoms, respectively.

**Methods:**

A total of 51 IBS patients were recruited and stratified into two groups: IBS-D patients received *C. butyricum* CBM588 (Butirrisan®), while IBS-C patients received *B. longum* W11 (Bowell®). Symptom severity was assessed using the Irritable Bowel Syndrome Severity Scoring System (IBS-SSS) before and after a 3-month intervention. Generalized linear models and regression analyses were used to evaluate treatment effects.

**Results:**

Both probiotic formulations significantly reduced IBS-SSS scores, particularly improving bloating, abdominal pain, and overall quality of life. The impact of treatment was independent of age, though greater improvements in bloating and life interference were observed in older IBS-C patients. A direct correlation between baseline symptom severity and symptom reduction was identified, suggesting higher efficacy in more severe cases.

**Conclusion:**

This study supports the use of *C. butyricum* CBM588 and *B. longum* W11 as effective probiotic interventions for IBS-D and IBS-C, respectively. Their strain-specific benefits highlight the potential of targeted probiotic strategies in IBS management. Future studies with larger sample sizes and longer follow-up periods are recommended to confirm and expand these findings.

## 1 Introduction

Irritable bowel syndrome (IBS) is a widespread chronic functional gastrointestinal disorder affecting individuals worldwide. In some developed countries, it is more commonly diagnosed in women than in men. The diagnosis relies on the ROME IV criteria, which define IBS by symptoms such as irregular bowel movements—ranging from constipation to diarrhea—abdominal pain or discomfort that eases after passing gas or stool, and the exclusion of underlying organic conditions like inflammatory bowel disease (IBD), celiac disease, and colorectal cancer (1, 2). Symptoms must have been present for at least 6 months before diagnosis and follow specific clinical patterns. IBS is categorized based on the dominant symptomatology into IBS with diarrhea (IBS-D), IBS with constipation (IBS-C), and a mixed type (IBS-M) characterized by alternating constipation and diarrhea (2, 3). Additionally, psychological factors significantly influence the onset, persistence, treatment-seeking behavior, and response to therapies (4–6).

The impact of IBS on the quality of life of affected individuals is not negligible. IBS is often associated with other comorbidities such as pain syndromes, migraine, depression, and visceral sensitivity. The impact of IBS on quality of life is considered comparable to that of other chronic diseases that are more widely recognized by the population, such as diabetes mellitus and hepatitis. Moreover, since there is currently no treatment capable of curing individuals affected by IBS, symptom improvement remains the most important goal. This is especially crucial because when symptoms improve, quality of life consequently improves, allowing affected individuals to lead a life as normal as possible (3). Among the various elements and strategies involved in the development and management of IBS, dietary, pharmacological, nutraceutical, and probiotic approaches have been proposed (1, 7, 8). In particular, nutraceuticals and botanical food supplements commonly used for weight management have been shown to exert prebiotic effects, modulating key bacterial populations such as *Bifidobacterium* spp., *Lactobacillus* spp., *Akkermansia muciniphila*, and *Faecalibacterium prausnitzi*, which are implicated in gut health and metabolic balance (9). These findings suggest that certain nutraceutical compounds may play a role in IBS management through microbiota modulation. Probiotic approaches have progressively shifted over the years from more generalist strategies to solutions increasingly tailored to specific objectives, introducing concepts such as bacterial therapy (10), bioprotic therapy, or precision probiotics (11). Recent studies have shown that multi-strain preparations containing *Lactobacillus* spp., *Enterococcus faecium*, and *Saccharomyces boulardii* can significantly reduce IBS symptoms, especially in patients with SIBO, improving abdominal pain, stool consistency, and overall satisfaction with intestinal function, although some methodological limitations have been acknowledged (12). Martoni et al. (13) evaluated the effects of *Lactobacillus acidophilus* DDS-1 and *Bifidobacterium lactis* UABla-12 on IBS symptoms. Both probiotics significantly improved abdominal pain, symptoms, and quality of life, with *Lactobacillus acidophilus* DDS-1 showing a higher response rate (52.3%) compared to *Bifidobacterium lactis* UABla-12 (28.2%). Additionally, both probiotics normalized stool consistency over time. Considering the natural evolution in the use of probiotics, the aim of this study is to investigate whether a specific, precision-based probiotic strategy, employing dedicated bacterial strains, can effectively manage symptoms in IBS-C and IBS-D patients, thus providing new insights into the real-life application of targeted microbiota modulation.

## 2 Probiotics in different bowel disorders

Probiotics, by modulating the intestinal microbiota, can be useful in various conditions, improving metabolic aspects, reducing subclinical inflammation, and positively influencing the onset and progression of oncological diseases. Similarly, nutraceuticals like berberine, through microbiota modulation, could complement these benefits (14). In addition, probiotics have garnered significant interest for the treatment of various intestinal conditions. They have shown effectiveness in reducing the duration of acute infectious diarrhea, whether bacterial or viral in origin, as well as in preventing traveler’s diarrhea. Studies have also highlighted benefits in treating acute diarrhea in children, with a significant reduction in the number of days of illness. Moreover, probiotic supplementation during *Helicobacter pylori* treatment can improve eradication rates, reduce side effects of the therapy, and alleviate associated clinical symptoms. However, the results should be interpreted with caution, given the heterogeneity among the studies included in this analysis (15). In this meta-analysis (16), 15 RCTs with 2,963 participants showed that Lactobacillus GG significantly reduces the duration of diarrhea (−1.05 days) compared to placebo. The best results were obtained with daily doses ≥10^10^ CFU, especially in patients treated in Europe. However, due to methodological limitations, the results should be interpreted with caution (17). Although probiotics have shown some potential in various clinical fields, current evidence does not support their use in the management of functional constipation in children: a recent review of randomized trials found no significant benefits compared to placebo or laxative monotherapy, highlighting the need for further studies to define optimal strains, doses, and treatment durations (18). Experimental evidence suggests that changes in the gut microbiome, caused by environmental or dietary factors, may play a role in the etiopathogenesis of IBD. Probiotics, such as *Lactobacillus* and *Bifidobacterium* strains, act by suppressing pathogen growth, modulating the immune system, and improving the intestinal barrier (19). A promising advancement in this area is the genetic engineering of *Escherichia coli* Nissle 1917 (ECN), which has been modified to overexpress antioxidant enzymes like catalase and superoxide dismutase, potentially enhancing its ability to treat intestinal inflammation and improve gut health (20, 21).

## 3 Probiotic therapy applied to IBS: from the constipation variant to the diarrhea variant

Among the various approaches proposed for managing IBS, the low FODMAP diet is currently one of the most effective strategies. Literature reviews encompassing several thousand patients, have demonstrated a significant reduction in associated symptoms and symptom severity (8, 22). In the absence of celiac markers, some IBS patients may benefit from a gluten-free diet (GFD). Although the mechanism is not clear, studies suggest that gluten may increase intestinal permeability in IBS-D. However, the reduction of fructans in the GFD could be the main cause of the observed benefits, making it as useful as the low FODMAP diet (23). In a systematic review, Ley et al., analyzed different intervention strategies and reported that a low FODMAP diet is associated with a symptom reduction of 70.8%, while probiotic treatment is associated with a reduction of 65.1%. The combination of a low FODMAP diet and probiotic treatment leads to an 80.4% reduction in symptoms. Considering the reduction in IBS-SSS, the low FODMAP diet is linked to a 90.5% decrease in the score, while probiotic treatment results in a 62.3% reduction, and the combination of both treatments leads to a 90.5% reduction, proving to be the most effective solution (22). Given these findings, the use of probiotics with a targeted approach in IBS treatment represents a particularly promising area of investigation. Moreover, rifaximin, a nonsystemic gut-selective antibiotic, has demonstrated efficacy in clinical trials for improving global symptoms, bloating, abdominal pain, and stool consistency in patients with IBS-D. Its effects on gut microbiota are modest and transient, suggesting symptom relief may involve mechanisms beyond microbial modulation (23, 24).

When considering the incidence of IBS in the two analyzed subtypes, it is important to discuss the crucial role of age: in the systematic review by Pittayanon et al. (25), a significant prevalence is observed in adult subjects (aged between 20 and 50 years), while only three studies included pediatric participants and two did not report the age of participants. It is also important to highlight that more than 40% of the included studies did not specify whether case and control groups were age-matched, thus limiting conclusions regarding the influence of age. Further data helping to better delineate the phenomenon can be found in the study by Wilkins et al. (26), which indicates that the peak incidence of IBS occurs between the ages of 20 and 39. This is consistent with the statements found in the Rome IV criteria, where the typical age of onset for IBS is between 20 and 40 years, with a progressive decline after age 50. It is also noted that, although less commonly diagnosed after age 60, the disorder may persist if previously established (27).

### 3.1 *Clostridium Butyricum* CBM588

*Clostridium butyricum* (CB) is a beneficial symbiotic bacterium, Gram-positive, butyrate-producing and obligate anaerobe, known for its ability to form spores. It is widely distributedin various environments, with a significant presence in the soil, and can also be detected in the human colon, where it ferments non-digestible carbohydrates to produce butyric acid. It is estimated to be present in approximately 20% of adults (28). This microorganism has attracted considerable scientific attention, as it is already widely used in some Asian countries, such as Japan, Korea, and China, as a safe and effective treatment for various gastrointestinal disorders, particularly persistent diarrhea and antibiotic-associated colitis (29). *Clostridium butyricum* (CB) is a probiotic used in several gastrointestinal diseases. A study by Zhao et al. assessed its efficacy in treating visceral hypersensitivity associated with IBS, demonstrating that its intake significantly reduces low-grade inflammation in the colonic mucosa through the regulation of the NLRP6 receptor, leading to a decrease in the expression of pro-inflammatory cytokines (IL-18, IL-1β) and other inflammatory markers. This process improves the intestinal barrier function and reduces visceral hypersensitivity (30). A prospective, multicenter, randomized, double-blind, placebo-controlled study further evaluated the efficacy and safety of CB in treating diarrhea-predominant IBS (IBS-D). The study analyzed 200 IBS-D patients treated with CB or placebo for 4 weeks, showing that CB administration effectively improved overall symptoms, quality of life, and bowel movement frequency compared to placebo (31). In particular, the CBM 588 strain (*Clostridium butyricum* MIYAIRI 588) has demonstrated notable positive effects, including the ability to stimulate mucin production to protect the intestinal barrier, strengthen epithelial tight junctions—crucial in preventing diarrhea—and modulate inflammatory and immune responses (32). To date, CBM 588 is the only *C. butyricum* strain cultivable on a large scale, supported by solid scientific evidence confirming its safety. The administration of this probiotic has proven particularly useful in managing gastrointestinal infections, especially those related to antibiotic use. Additionally, numerous studies highlight its ability to counteract infections caused by common pathogens such as *Escherichia coli, Helicobacter pylori, Staphylococcus aureus,* and *Salmonella* spp. (33). Regarding its role in managing diarrhea, substantial clinical data support its effectiveness. One of the most significant studies in this field is a 2017 randomized, double-blind, placebo-controlled clinical trial on patients with inflammatory bowel disease (IBD), which examined its efficacy in controlling IBD-related diarrhea with highly positive outcomes (34). Specifically analyzing CBM 588 in the treatment of irritable bowel syndrome (IBS) and its impact on gut microbiota, a study involving 30 IBS patients who received *C. butyricum* CBM 588 for 14 days showed a decrease in the average number of daily bowel movements from 6.0 ± 5.6 to 1.7 ± 1.1 (*p* < 0.001), with an overall response rate of 83.4%. Improvement in diarrhea was observed in 86.4% of patients from the first day of treatment, with no reported side effects. Additionally, *C. butyricum* promoted the growth of beneficial bacteria such as *Bifidobacterium* and *Lactobacillus*, while reducing the concentration of pathogenic bacteria and restoring a balanced gut microbiota, all without side effects (34). In summary, the combination of mechanisms supporting the use of CBM588 as a precision probiotic tool for managing IBS-D includes, as previously discussed, its ability to produce butyrate *in situ*, providing an energy substrate for colonocytes, supporting the function of tight junctions and the intestinal barrier, and exerting a significant anti-inflammatory effect (31). Moreover, as described by Ariyoshi et al., CBM588 stimulates the production of γδ T cells and CD4 + T cells that secrete interleukin-17A (IL-17A), while also promoting the activation of B cells and the production of immunoglobulin A. Additionally, it enhances the production of palmitoleic acid, 15-deoxy-prostaglandin J₂, and protectin D1, and stimulates the proliferation of Bifidobacteria and Lactobacilli. These effects contribute positively to immune and mucosal function, supporting the modulation of pathophysiological processes and symptoms underlying IBS-D (35).

### 3.2 *Bifidobacterium longum* W11

*Bifidobacterium longum* W11 (LMG P-21586) was isolated from a healthy human donor and deposited in the DDBJ/EMBL/GenBank database (36). The strain has been extensively studied for its resistance and adaptability within the gastrointestinal tract, demonstrating a high survival rate (approximately 80%) and notable growth capacity (around 55%) at a pH like that of the gastric environment (37). It also exhibits good adhesion capabilities under simulated intestinal conditions, which have been confirmed *in vivo* through its ability to produce exopolysaccharides (38). Its interaction with peripheral blood mononuclear cells (PBMCs) is associated with a Th1-type immune response, an increase in IFN-*γ* and IL-2, and a concurrent reduction in IL-10, without causing excessive inflammatory effects, as demonstrated by the absence of TNF-*α* induction (8). One of the primary clinical benefits of W11 colonization is of its ability to improve constipation, particularly in patients diagnosed with IBS-C. A clinical trial involving 636 IBS-C patients who received 5 billion CFU per dose showed a 25% increase in intestinal motility. The average number of weekly bowel movements increased from 2.9 to 4.1, with 80% of participants reporting improvement at the end of the study, and 60% maintaining positive effects even after treatment discontinuation (39). Summarizing the effects described in the literature for the W11 strain in the context of IBS-C treatment, Inturri et al. report the production of exopolysaccharides (EPS), which may contribute to the formation of protective biofilms on the intestinal mucosa and promote an immuno-epithelial environment (38), supportive of IBS-C management. This interesting effect is complemented by the strain’s ability to produce the enzyme arabinofuranosidase—encoded by the *abfA* and *abfB* genes, which are overexpressed in W11—enabling it to metabolize arabinans found in plant-derived foods. This metabolic activity leads to the production of acetate and butyrate, which, through interaction with specific receptors such as GPR41 (FFAR3) (40) and GPR43 (FFAR2) (41), stimulate intestinal motility, modulate inflammatory status, and improve stool consistency (42).

Visual scale assessments highlighted a significant reduction in bloating and abdominal pain. The percentage of participants without bloating symptoms increased from 3 to 26.7%, while those without abdominal pain rose from 8.4 to 44.1%. Among individuals with moderate-to-severe symptoms, the frequency significantly decreased from 62.9 to 9.6% for bloating and from 38.8 to 4.1% for abdominal pain. Another study conducted on 129 IBS-C patients confirmed these findings, showing an increase in weekly bowel movements (from approximately 14 to 17) and a 40% reduction in gastrointestinal symptoms, including abdominal pain and bloating (43). Similar benefits have also been demonstrated in individuals suffering from constipation unrelated to IBS-C. In a study evaluating 300 women with hypocaloric diet-induced constipation, treatment with *B. longum* W11 led to a statistically significant increase in bowel movement frequency in approximately 30% of participants (44).

Di Pierro et al. (45) evaluated the efficacy of the concomitant administration of *Bifidobacterium longum* W11 and rifaximin in patients with Uncomplicated Symptomatic Diverticular Disease (SUDD), compared to the traditional strategy in which the probiotic was taken after the antibiotic. The study analyzed whether this combination improved therapy adherence and symptom control. The choice of the *B. longum* W11 strain was due to its non-transferable resistance to rifaximin, making it suitable for simultaneous use with the antibiotic.

It can be hypothesized that the administration of *B. longum* W11 alongside rifaximin contributed to improving clinical outcomes by limiting factors that could compromise gut microbiota balance and preventing damage to the bacterial consortium.

The aim of this study is to evaluate the effects of *Clostridium butyricum* CBM588 and *Bifidobacterium longum* W11 in the management of IBS-D and IBS-C, respectively, to assess their actual clinical response capacity in a real-life setting. Both supplements have been on the market in Italy for a long time, *Clostridium butyricum* CBM588 is marketed in Italy by Pharmextracta SPA as Butirrisan®; (registered with the Italian Ministry of Health under the number 152311), and *Bifidobacterium longum* W11 is marketed in Italy by Pharmextracta SPA as Bowell®; (registered with the Italian Ministry of Health under the number 92629).

## 4 Materials and methods

### 4.1 Study design and participants

This study involved a total of 51 participants diagnosed with IBS were recruited and stratified based on their predominant symptom type. Subjects with diarrhea-predominant IBS (IBS-D) received CBM588, while those with constipation-predominant IBS (IBS-C) were administered W11. The study followed a prospective, repeated-measures design with a pre-treatment and post-treatment evaluation. Participants in the CBM588 group (*n* = 24) took three tablets per day for three consecutive months, whereas those in the W11 group (*n* = 27) consumed one stick per day for the same duration. The study protocol was approved by the Ethics Committee for Human Experimentation of Urbino (nr. 90, January 23, 2025), and all participants provided informed consent before enrolment.

The demographic characteristics of the sample analyzed show good consistency with those reported in the literature and previously discussed (onset typically between 20 and 40 years, with a progressive decline after 50, and persistence beyond 60 years if already present in earlier decades) (25–27). As shown in Table 1, the mean age of the IBS-D group was 54.4 ± 19.8 years, while the IBS-C group had a mean age of 55 ± 20.2 years. When considering the higher age brackets, it is important to note that in almost all cases, the patients presented with the condition from an earlier age range consistent with that described in the literature.

**Table 1:** Demographic characteristics of Butirrisan and Bowell groups stratified by sex. Age is reported as mean ± SD.

Sex	*n* and Age	Butirrisan	Bowell	*p* value
Males	*n*	12	14	
	Age (years)	62.7 ± 18.1	56.3 ± 20.3	0.41
Females	*n*	12	13	
	Age (years)	46.2 ± 18.5	54.2 ± 20.9	0.32

### 4.2 Outcomes measures

The primary outcome measure was the Irritable Bowel Syndrome Severity Scoring System (IBS-SSS), a validated questionnaire assessing the severity of IBS-related symptoms. The questionnaire evaluates five symptom domains: abdominal pain intensity, abdominal pain frequency, bloating, dissatisfaction with bowel habits, and interference with daily life. Each item is scored, and the total IBS-SSS score categorizes symptom severity into mild (75–174), moderate (175–299), and severe (>300).

### 4.3 Statistical analysis

To assess the effectiveness of the supplements, a generalized linear model (GLM) for repeated measures was employed. This statistical analysis was conducted separately for W11 and CBM588 supplements. This model accounts for within-subject correlations across time points and allows for the analysis of changes in IBS-SSS scores. Time (pre-treatment vs. post-treatment) was included as a within-subject factor, while age was analyzed as a covariate to evaluate its potential moderating effects. Effect sizes were quantified using Cohen’s *d*, interpreted as follows: 0.2 (small), 0.5 (medium), and 0.8 (large). Between-group comparisons were performed using independent sample *t*-tests, while within-group changes were analyzed using paired *t*-tests. Additionally, linear regression analyses were conducted to explore the relationships between age, pre-treatment symptom severity, and the extent of symptom improvement. All statistical analyses were conducted using R software (version 4.4.2), and a significance threshold of *p* < 0.05 was applied for all tests.

## 5 Results

A total of 24 subjects with diarrhea-predominant IBS (IBS-D) took the supplement CBM588 at a dosage of 3 tablets per day for 3 consecutive months, while 27 subjects with constipation-predominant IBS (IBS-C) took 1 stick of the supplement W11 for the same period. The group treated with CBM588 had a mean age of 54.4 ± 19.8 years (min-max 23–86), while the group treated with W11 had a mean age of 55.3 ± 20.2 years (min-max 18–85). Differences between the two groups were tested using an independent samples *t*-test, and no statistically significant differences were identified in any case, allows us to perform comparisons between the two groups. The sample characteristics are reported in Table 1.

The mean differences between post- and pre-treatment were first evaluated for each individual supplement and were assessed both in relation to the individual items and the final score of the IBS-SSS questionnaire. Using GLM, time effect was statistically significant for all scores, indicating a clear reduction in IBS severity scores in the post-treatment period. These results are reported in Table 2, where Cohen’s *d* coefficient for the estimate Effect Size is interpreted as follows: 0.2 small, 0.5 medium, 0.8 large (46).

**Table 2:** Mean differences of single items of IBS-SSS and total score, evaluated between after and pre-scores for each supplement (Butirrisan and Bowel in this table are evaluated separately). In each box is reported the *p* value obtained by a *t* test for paired data, with a significance level fixed at 0.05. in the last column are indicates Choen’s d coefficient for the effect size.

Item considered	Supplement	Δ Mean	[S. E.] (*p*)	Cohen’s *d*
Δ Abdominal pain intensity	Butirrisan	−28.3	3.9 *(< 0.001)*	0.45
	Bowell	−17.1	2.9 *(< 0.001)*	0.31
Δ Abdominal pain frequency	Butirrisan	−20.0	3.6 *(< 0.001)*	0.33
	Bowell	−10.3	3.1 *(< 0.01)*	0.18
Δ Bloating	Butirrisan	−30.8	3.4 *(< 0.001)*	0.55
	Bowell	−25.9	2.9 *(< 0.001)*	0.41
Δ Dissatisfaction of bowel habit	Butirrisan	−20.8	4.3 *(< 0.001)*	0.31
	Bowell	−9.5	3.8 *(< 0.05)*	0.14
Δ Life-interference	Butirrisan	−17.5	2.1 *(< 0.001)*	0.48
	Bowell	−11.1	2.0 *(< 0.001)*	0.27
Δ Ibs-sss total score	Butirrisan	−117.5	12.9 *(< 0.001)*	0.56
	Bowell	−74.0	9.47 *(< 0.001)*	0.38

**Figure 1:**
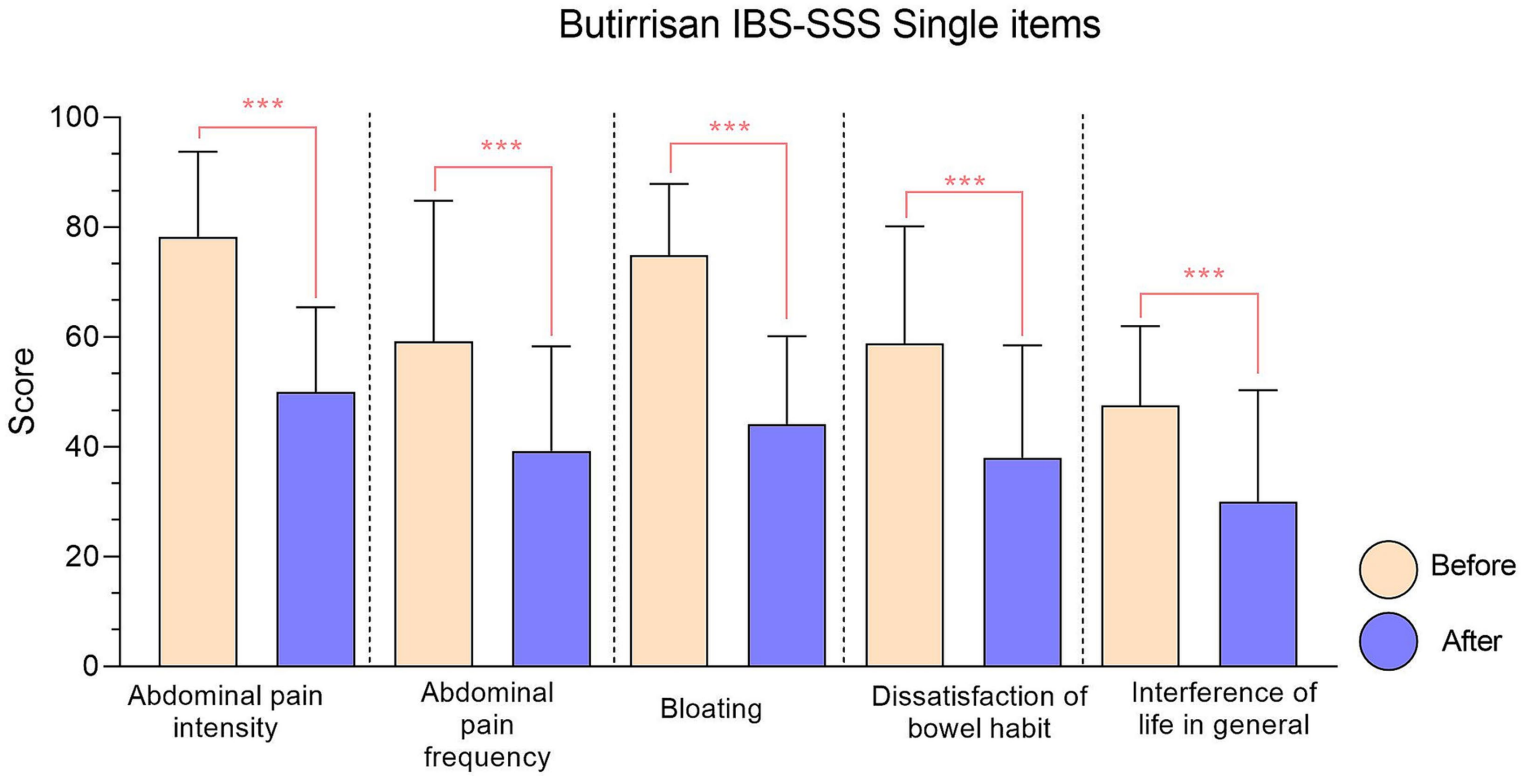
IBS-SSS single items relative to Butirrisan® supplement. Every item reduction *results* significant, with a *p* value < 0.01. In all graphs, significance level is represented with: * (*p* < 0.05); ** (*p* < 0.01); ***(*p* < 0.001).

**Figure 2:**
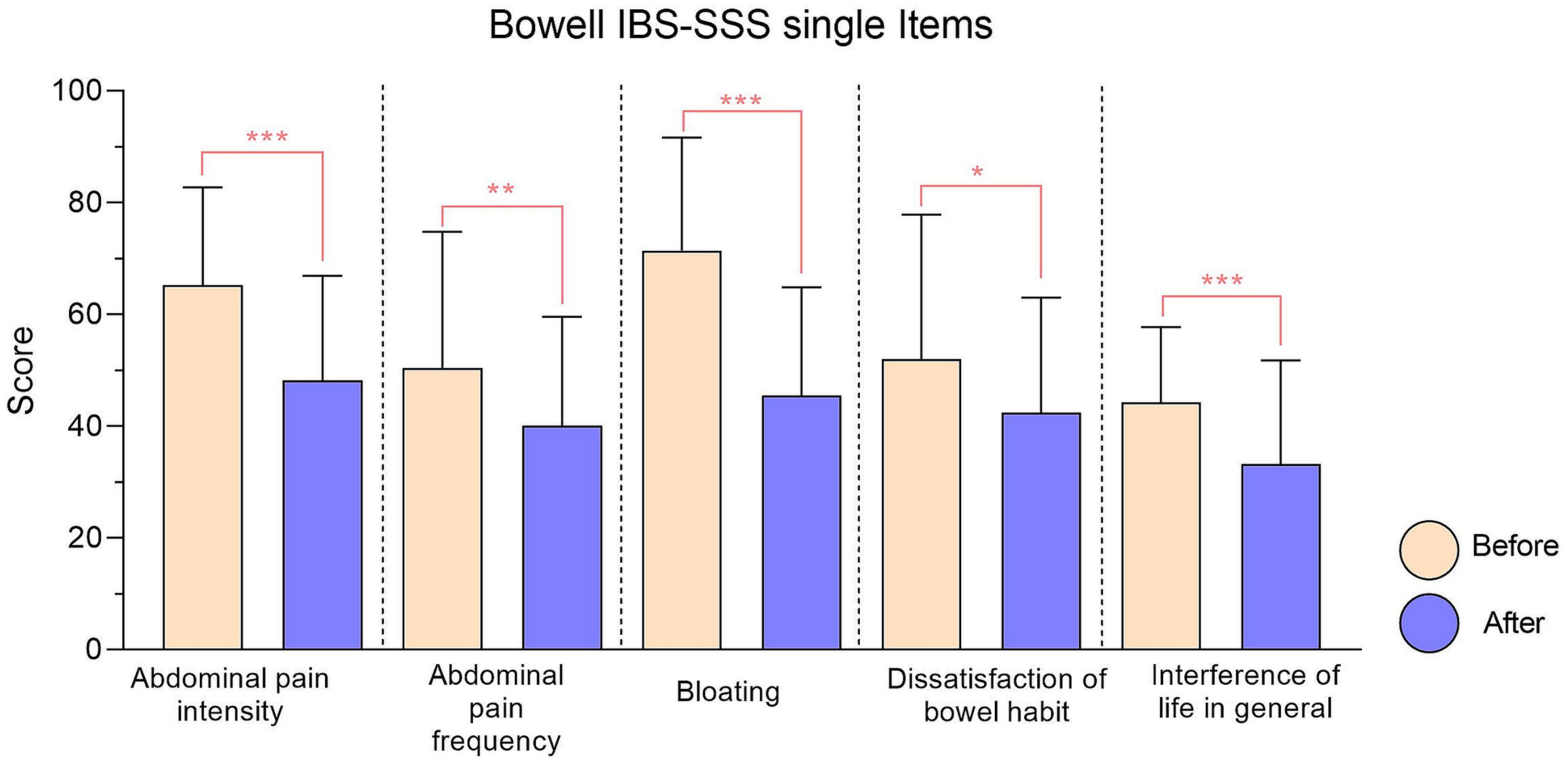
IBS-SSS single items relative to Bowell® supplement. Every item reduction *results* significant. Significance is represented with: * (*p* < 0.05); ** (*p* < 0.01); ***(*p* < 0.001).

To gain an even clearer view of the decrease in the score for each individual item, the data presented have been gathered in Figures 1, 2.

**Figure 3:**
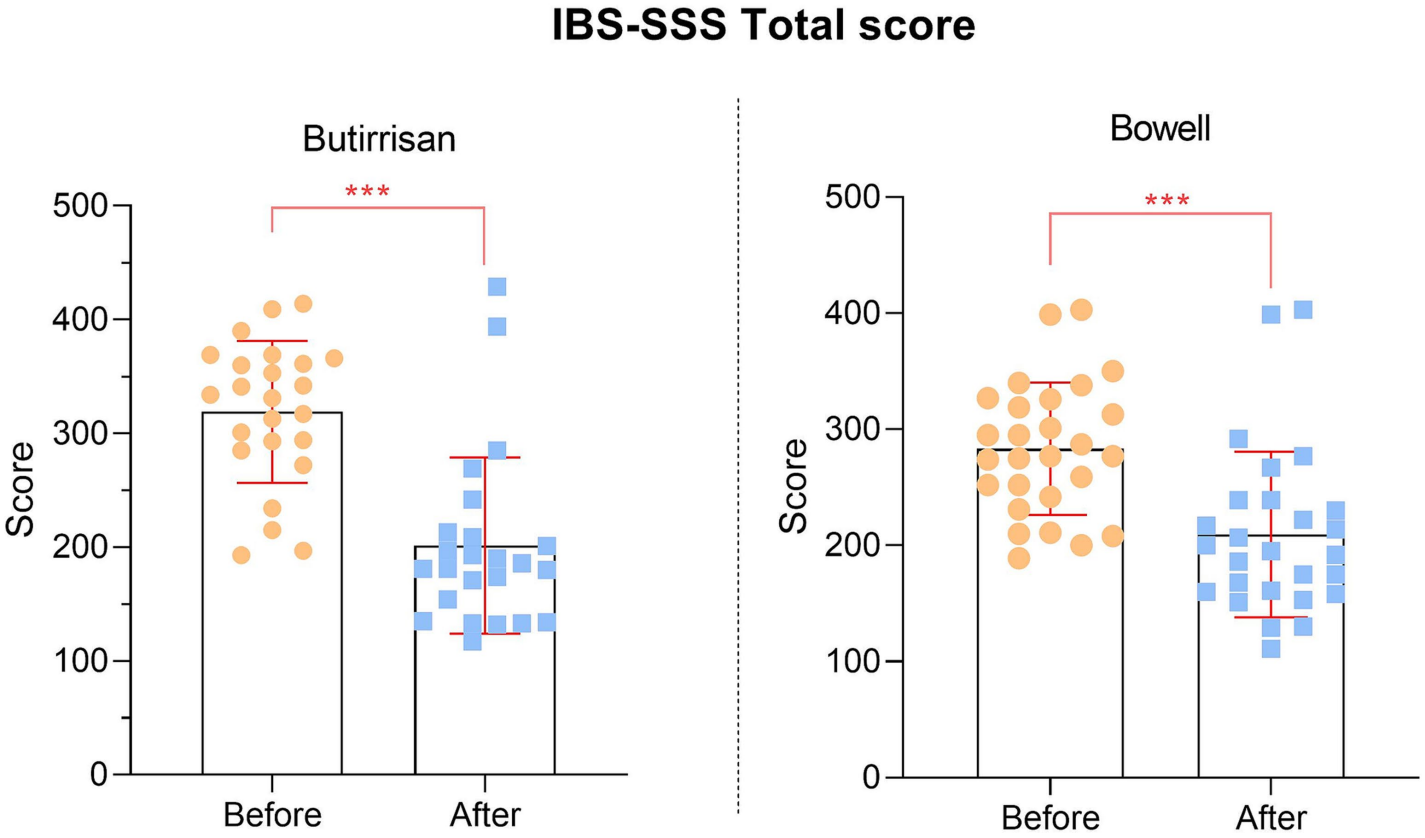
IBS-SSS total score reduction for each supplement.

Since all individual items of the SSS questionnaire are significant, the same applies to the final scores, presented in Figure 3.

Regarding the W11 supplement, in addition to the significance observed between pre- and post-treatment scores, a significant effect of time * age was identified for the variables: bloating (*F* = 8.36, *p* = 0.008), interference with life in general (*F* = 6.40, *p* = 0.02), and IBS-SSS total score (*F* = 7.56, *p* = 0.01). This indicates that the evolution of bloating symptoms, interference with daily life, and the total score over time varies depending on the subjects’ age. The impact of the time factor, and thus the improvements following treatment in relation to these variables, differs based on age. Regarding the CBM588 supplement, a significant effect of the time on the following variables: bloating (*F* = 4.63, *p* = 0.04), interference with life in general (*F* = 6.40, *p* = 0.02), and total score (*F* = 4.73, *p* = 0.04), indicating a significant influence of time on these variables, suggesting a beneficial effect of the CBM588 supplement. Interesting between-subject effect for age was found on abdominal pain frequency (*F* = 12.17, *p* = 0.002), Bloating (*F* = 16.17, *p* = 0.001), dissatisfaction of bowel habits (*F* = 10.72, *p* = 0.004), total score (*F* = 15.40, *p* = 0.001). Indicating that these variables are significantly influenced by age, independently of the treatment and time.

**Figure 4:**
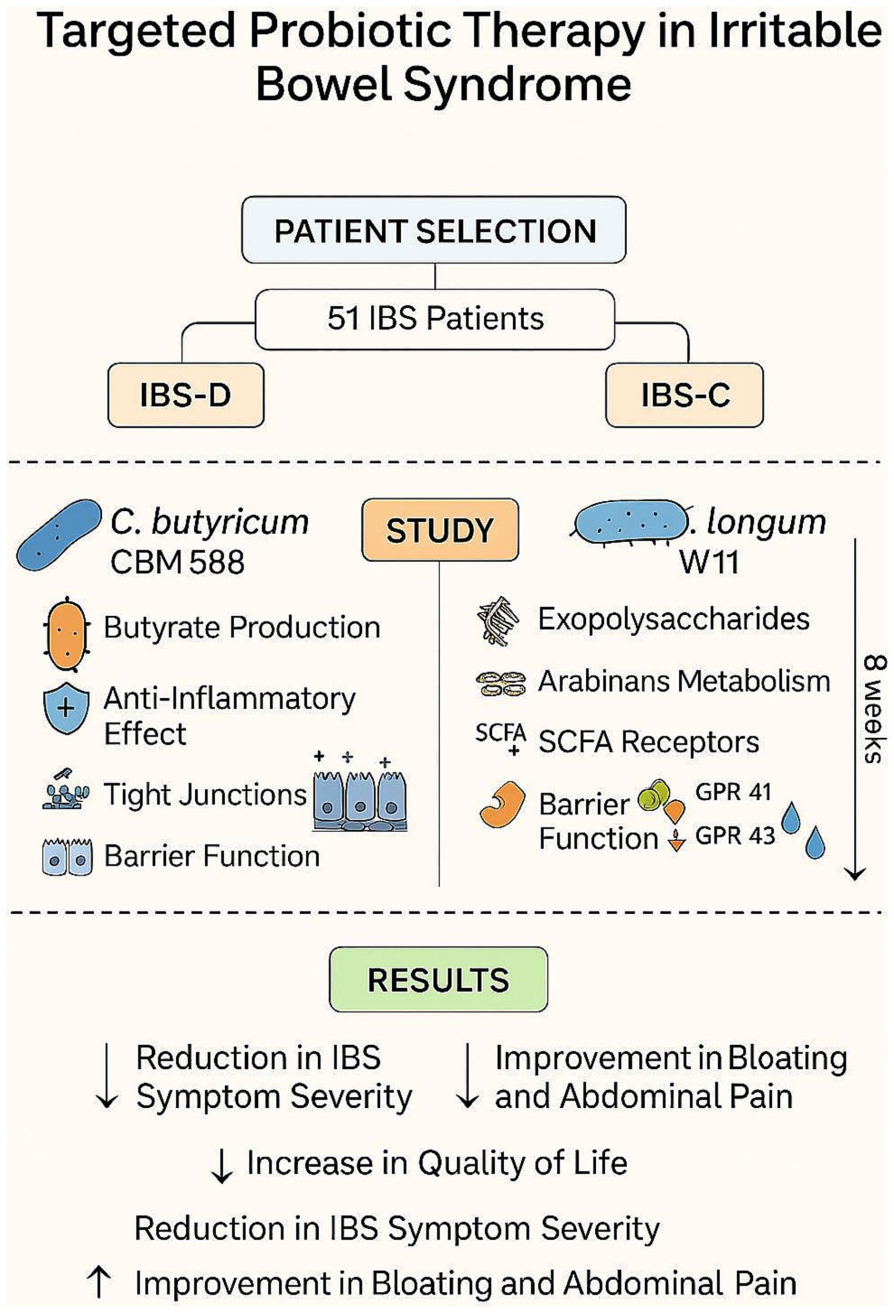
Regression lines of age vs. pre-treatment score for Butirrisan® and Bowell®. Significance is represented with * (*p* < 0.05); ** (*p* < 0.01); ***(*p* < 0.001).

Figure 4 provides a schematic summary of the study design and the main findings.

### 5.1 Regressions

**Figure 5:**
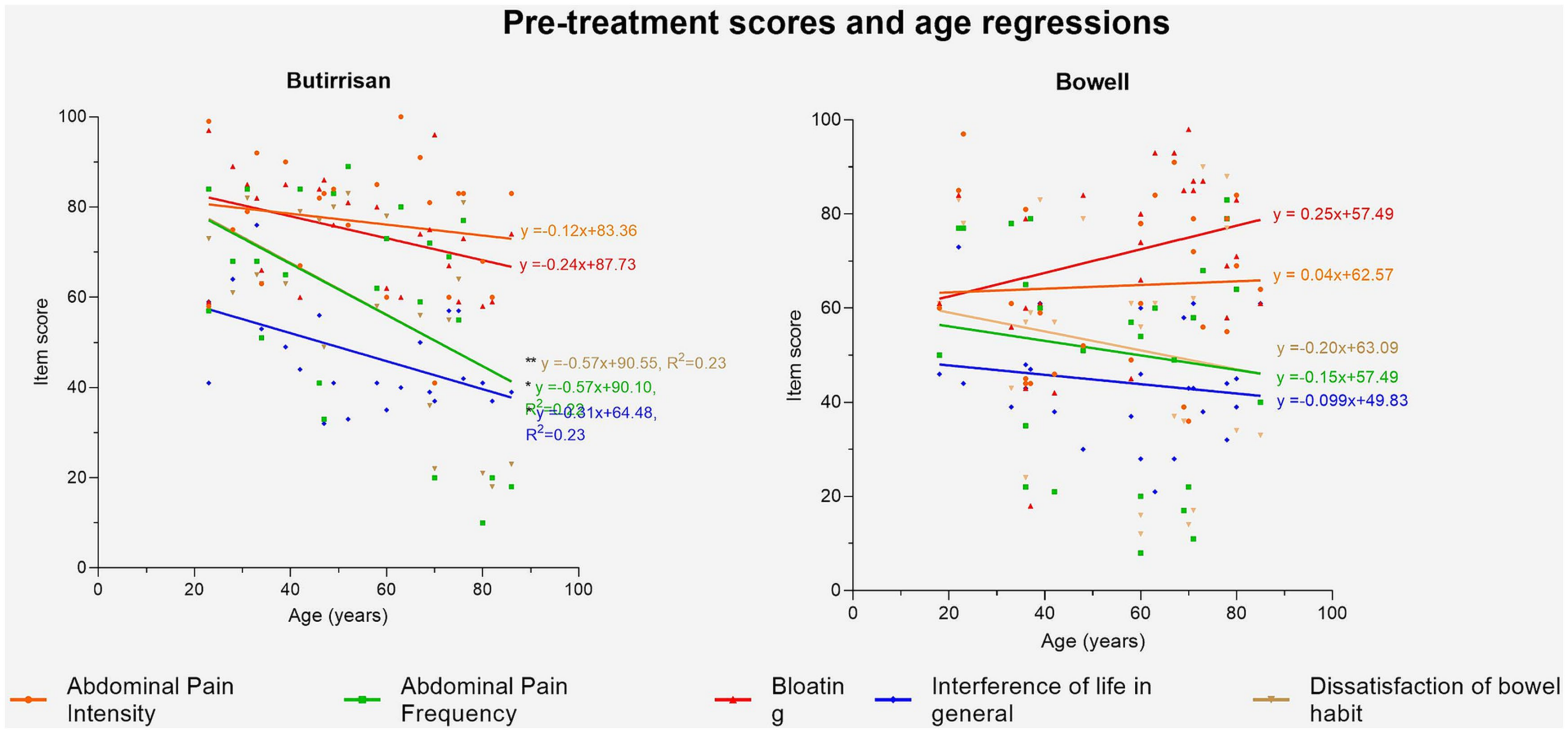
Regression lines of age vs. score change delta. Significance is represented with: * (*p* < 0.05); ** (*p* < 0.01); ***(*p* < 0.001).

Given the significant influence of age on some of the variables in the IBS questionnaire, the impact of age in greater depth, both on the perception of various symptoms before the intervention and on the variation of individual scores were explored (Figure 5). The first aspect was analyzed by performing a linear regression between age (independent variable) and the T0 score of individual items for both supplements. The results were interesting. In the case of CBM588 (administered to subjects with the diarrhea variant), symptoms were found to be more debilitating in younger individuals. The regression lines concerning dissatisfaction with bowel habits, the frequency of abdominal pain, and interference with daily life were found to be statistically significant, indicating that the perception of these symptoms is significantly higher (*p* < 0.05) in younger individuals compared to adults and the elderly. The same analysis was conducted for the W11 supplement. In this case, no correlation was found to be significant, and only the perception of bloating appeared to increase with age, although it did not reach the level of significance (*p* = 0.19).

**Figure 6:**
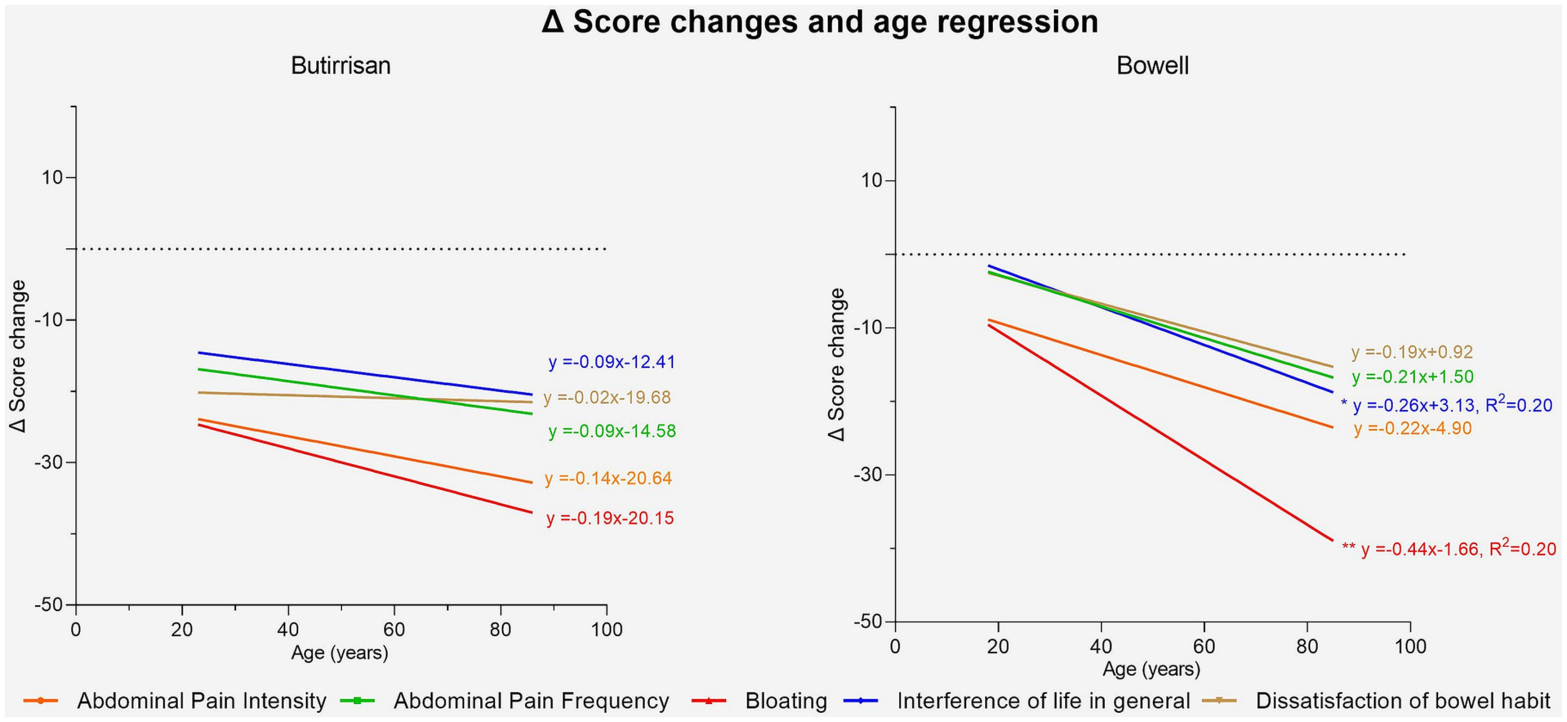
Regression lines of individual items between pre-treatment score and post-pre change delta for the Butirrisan® supplement. All regression lines are highly significant, indicating that a worse initial condition may result in greater effectiveness of the supplement.

Using linear regression, it was then investigated whether the improvement in individual scores could depend on age. Regarding CBM588, a systematic effect of the supplement in reducing symptoms emerged, regardless of age, and no regression line was found to be statistically significant. As for W11, age was found to be correlated with a greater reduction in two specific symptoms following the intake of the supplement: bloating (*p* < 0.01) and interference with daily life (p < 0.01), indicating greater benefits in the adult-elderly population. These results are shown in Figure 6.

**Figure 7:**
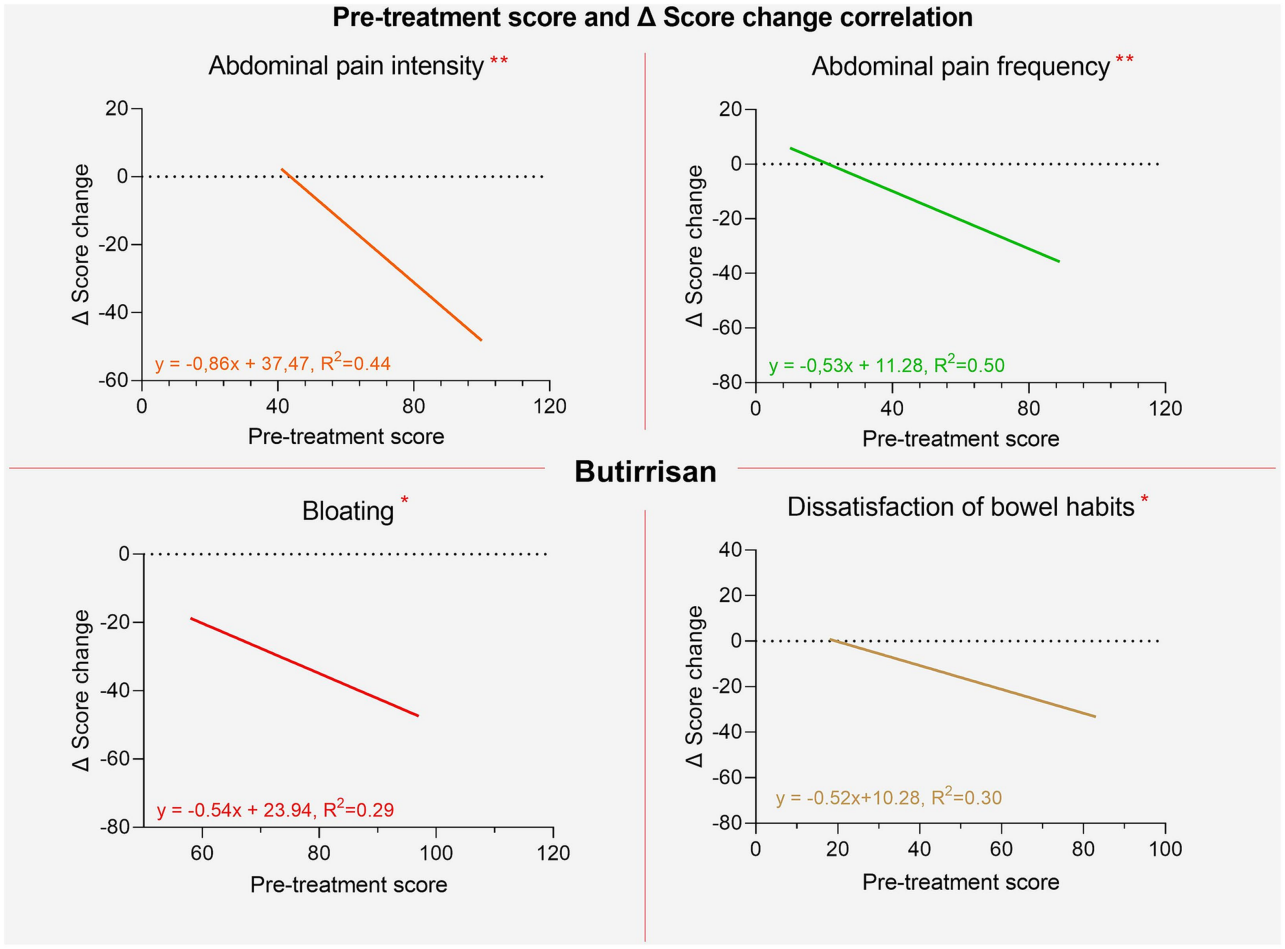
Regression lines of individual items between pre-treatment score and post-pre change delta for the Bowell® supplement.

**Figure 8:**
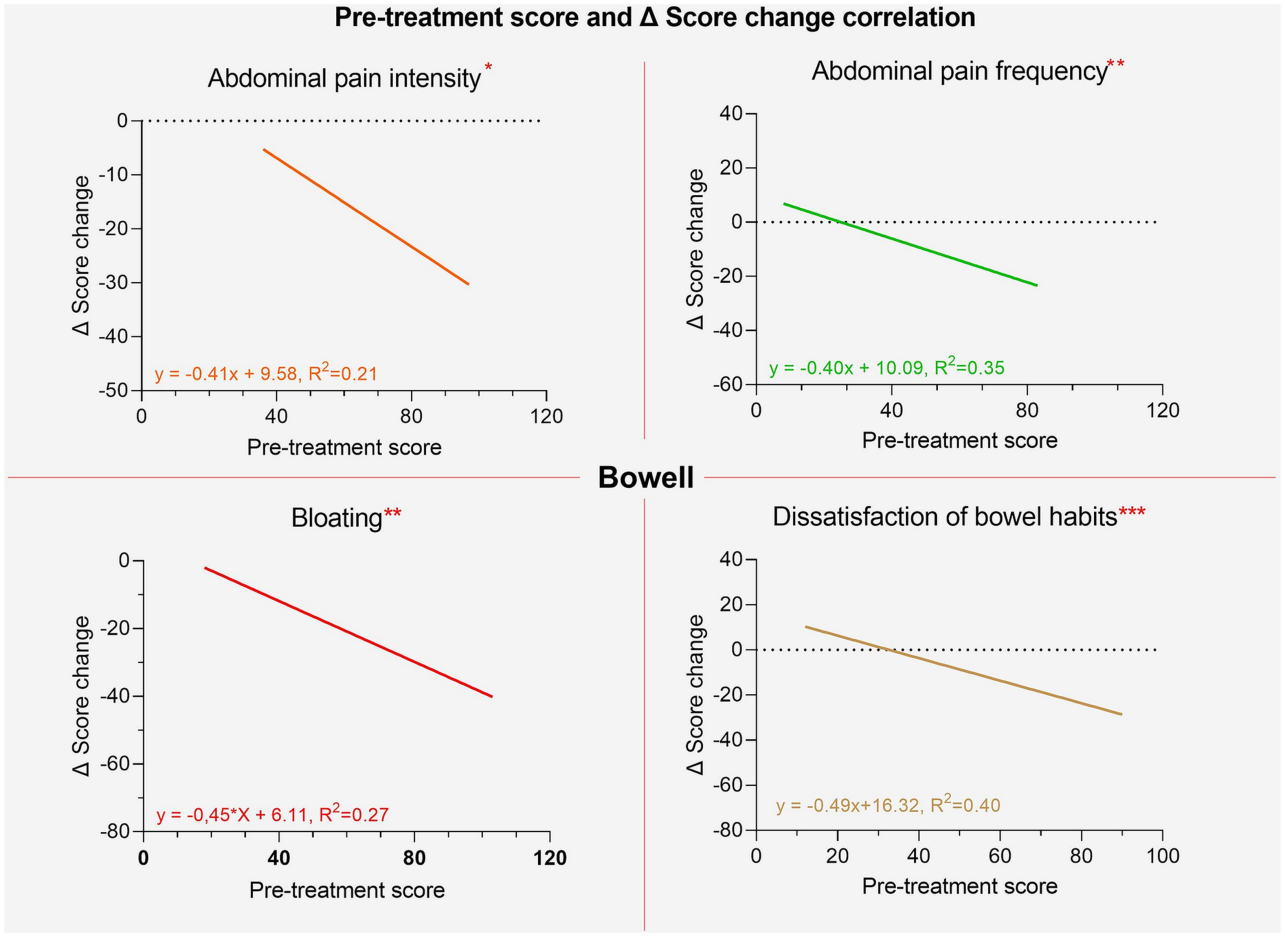
Regression lines of individual items between pre-treatment score and post-pre change ∆ for the Bowell supplement.

It was also investigated whether there was a correlation between the initial scores and the extent of improvements following the intake of the supplements. For both supplements, significant correlations were found for all variables except for interference with daily life. This suggests that the worse the initial severity of symptoms, the greater the extent of improvement, measured as the difference in individual item scores between the two phases (pre and post). These results are shown in Figures 7, 8.

For the CBM588 supplement, we can observe a 33% increase in mild cases and a 29% increase in moderate cases, alongside a 63% reduction in severe cases. For the W11 supplement, severe and moderate cases were reduced by 30 and 7%, respectively, while the number of subjects with mild severity increased by 37% (Table 3). In addition, each participant was asked to report any side effects experienced during the period of probiotic intake, and none reported any adverse effects.

**Table 3:** The severity conditions of the IBS-SSS (Irritable Bowel Syndrome Severity Scoring System) questionnaire before and after treatment.

Supplement	Time	Mild *n* (%)	Moderate *n* (%)	Severe *n* (%)
Butirrisan	Pre	0 (0%)	7 (29%)	17 (21%)
	Post	8 (33%)	14 (58%)	2 (8%)
Bowell	Pre	0 (0%)	17 (63%)	10 (37%)
	Post	10 (37%)	15 (56%)	2 (7%)

## 6 Discussion

In this study, we analyzed the efficacy of *Clostridium butyricum* CBM588 and *Bifidobacterium longum* W11 in managing IBS in its IBS-D and IBS-C variants, respectively. Both strains demonstrated good efficacy in alleviating symptoms and improving clinical scores, without significant side effects. A key innovative aspect of this study is the approach that considers the dual nature in which IBS can manifest itself. One of the firsts findings concerns the relationship between age and symptom perception prior to treatment. In the group treated with CBM588, regression analysis showed that younger individuals reported more pronounced symptoms—such as dissatisfaction with bowel habits, more frequent abdominal pain, and greater interference with daily life—which tended to decrease with age (Figure 4). This may be explained by lifestyle differences: IBS-D may be more disruptive for younger individuals who typically lead busier lives and spend more time away from home, increasing their perception of discomfort.

In contrast, within the group treated with W11, elderly participants experienced significant improvements in bloating (*p* < 0.01) and interference with daily life (*p* < 0.05). The greater reduction in bloating was anticipated, as this symptom tends to be more pronounced with age, as shown in Figure 4 and supported by regression analysis comparing pre-treatment scores with symptom change (delta scores). For both supplements, the reduction in symptom scores after treatment was strongly correlated with baseline severity (Figures 6, 7). This indicates that a higher pre-treatment score results in a greater improvement in symptom perception, leading to a reduction in the interference of IBS symptoms with daily life and overall quality of life. This highlights how efficacy is closely correlated with the initial severity of symptoms (as indicated in Figures 6, 7). Interest in this type of intervention is growing, with several studies exploring the role of probiotics and dietary strategies in IBS management. These approaches act both at the organ level and on the gut microbiota, generating substantial data suitable for reviews and meta-analyses (7, 22). IBS, when correctly diagnosed according to Rome IV criteria, affects 3–5% of the global population and significantly impacts quality of life, healthcare resource use, and productivity (47). Approaches that target the gut microbiota are especially relevant, given its role in modulating immune and inflammatory responses and preserving intestinal epithelial function (48, 49). Maintaining a balanced microbiota, particularly with appropriate levels of *Firmicutes*, *Bacteroidetes*, *Actinobacteria*, and *Proteobacteria* (49) is crucial, along with the presence of beneficial strains such as *Lactobacilli* and *Bifidobacteria* (50). Recent studies have highlighted the microbiota’s involvement in intestinal motility, visceral sensitivity, and even brain function, underscoring a bidirectional gut-brain communication pathway involving immune, metabolic, and neurological signals (51). These mechanisms align with the known effects of *C. butyricum* CBM588 and *B. longum* W11, especially in IBS patients who often exhibit reduced microbial diversity, increased pro-inflammatory species, and decreased populations of anti-inflammatory, butyrate-producing bacteria (52). Notably, IBS-D has also been associated with small intestinal bacterial overgrowth (SIBO) (53). The ability to produce butyrate and SCFAs is particularly interesting for restoring epithelial integrity, as their levels are linked to the increased expression of tight junction proteins such as occludin and claudins and the production of E-cadherin (50, 54). This may explain the clinical benefits observed in IBS-D patients treated with *C. butyricum* CBM588. Regarding *Bifidobacteria*, numerous strains have shown promising results in IBS management. Benefits include the regulation of *μ*-opioid and cannabinoid receptor expression, which contribute to reduced bloating, improved transit time and evacuation frequency, pain relief, better global symptom control in IBS-C, and improved quality of life (33, 47, 55). These findings are consistent with the results observed in our study with *B. longum* W11. Lastly, a final consideration should be made regarding the reduction in total IBS symptom scores. Both probiotic treatments resulted in statistically significant reductions (*p* < 0.01), and beyond statistical significance, meaningful clinical improvements were also observed. However, a more detailed assessment of symptom severity is essential. According to standard scoring criteria, severity is defined as follows: 75–174 = mild, 175–299 = moderate, and >300 = severe (56). Overall, the results of our study are in line with the existing literature on similar probiotic strains and support a more targeted use of specific microbial agents for managing distinct IBS variants. A key strength of this study lies in its innovative design, which addresses the two predominant subtypes of irritable bowel syndrome, IBS-D and IBS-C, through a targeted and differentiated probiotic intervention. By evaluating *Clostridium butyricum* CBM588 and *Bifidobacterium longum* W11 in patient groups stratified by symptom profile, the study adopts a precision approach that mirrors the growing need for individualized treatment strategies in functional gastrointestinal disorders. Unlike many previous investigations that consider IBS as a single, homogeneous entity, this study highlights how distinct pathophysiological mechanisms may respond to specific microbial strains. The age-related differences in symptom perception, the correlation between baseline symptom severity and treatment response, and the absence of significant side effects further enhance the study’s relevance. Overall, the findings support the idea that precision probiotic therapy represents a valuable addition to the therapeutic arsenal for IBS, offering a tailored, microbiota-targeted strategy that aligns with the broader evolution of personalized medicine in gastroenterology.

### 6.1 Limitation

The number of subjects evaluated represents the first limitation of this study; observing a larger sample could contribute to obtaining even more solid results. Additionally, a longer observation period could prove useful in clarifying whether the described effects are long-lasting. Moreover, a study design that allows for monitoring against a control group could provide greater clarity regarding the extent of the observed data. Future studies might consider including a control group to rule out the influence of other confounding factors, like diet and comorbid conditions. It is also important to emphasize that recruiting the subjects within the authors’ clinical practice setting may limit the generalizability of the results to other populations.

## 7 Conclusion

This study demonstrated that *Clostridium butyricum* CBM588 and *Bifidobacterium longum* W11 are effective in managing IBS-D and IBS-C, respectively, significantly improving symptom severity and quality of life without notable side effects. The results highlight a direct correlation between symptom severity and treatment efficacy, reinforcing the potential of targeted probiotic interventions in IBS management. The observed improvements, particularly in bloating and overall symptom scores, align with existing literature, supporting the rationale for strain-specific probiotic strategies, also to improve the impact of symptoms on quality of life at any age.

## Data Availability

The raw data supporting the conclusions of this article will be made available by the authors, without undue reservation.
